# Tracheal mucosal necrosis and sloughing after accidental endotracheal extubation in a child: a case report and literature review

**DOI:** 10.3389/fped.2026.1715950

**Published:** 2026-02-05

**Authors:** Yanting Zhang, Luobei Zhang, Kang Xu, Jun Chen, Ying Xu, Yihua Yang, Jing Ma, Chang Liu

**Affiliations:** 1 Department of Critical Care Medicine, Zhongnan Hospital of Wuhan University, Wuhan, China; 2 Clinical Research Center of Hubei Critical Care Medicine, Wuhan, China

**Keywords:** case report, children, endotracheal intubation, obstructive fibrinous tracheal pseudomembrane (OFTP), tracheal injury

## Abstract

**Introduction:**

Airway obstruction caused by the detachment of obstructing fibrinous tracheal pseudomembrane (OFTP) secondary to airway mucosal injury from endotracheal intubation is extremely rare in clinical practice. Due to the non-specific clinical manifestations and low incidence of this disease, most clinicians tend to overlook it, lack vigilance, and find it difficult to make a definite diagnosis immediately when symptoms appear. After the tracheal mucosa is injured and detached, it can form a valvular obstruction that blocks airway ventilation, leading to obvious dyspnea, acute respiratory distress, and even asphyxia resulting in sudden death of the patient. Early definite diagnosis and correct management are conducive to turning the patient's critical condition around and promoting smooth recovery.

**Case presentation:**

We report a case of a pediatric patient who developed acute airway obstruction due to the necrosis and detachment of OFTP after accidental self-extubation of the endotracheal tube following intubation. Before a definite diagnosis was made, non-invasive ventilator-assisted ventilation and nebulization therapy were administered. After the diagnosis was confirmed by flexible bronchoscopy and cervical computed tomography, the necrotic airway mucosal tissue was removed via flexible bronchoscopy. The patient's airway obstruction was successfully relieved, symptoms of inspiratory dyspnea were alleviated, airway patency was restored, and the patient was eventually cured and discharged from the hospital.

**Conclusions:**

OFTP caused by accidental endotracheal extubation is a rare clinical condition that leads to acute inspiratory dyspnea. Flexible bronchoscopy is beneficial for diagnosis, as well as for non-invasive and rapid removal of necrotic and detached tracheal mucosal tissue formed by OFTP. This procedure relieves airway obstruction in patients and exerts a positive impact on the diagnosis, treatment, and prognosis of the patients.

## 1 Introduction

Endotracheal intubation (Endotracheal Tube, ET) is a crucial method for providing safe assisted ventilation to patients admitted to the Intensive Care Unit (ICU). Inevitably, ET can also cause a range of complications, including tracheal stenosis, circumferential tracheitis, granulomas, tracheomalacia, tracheoinnominate artery fistula, and tracheoesophageal fistula (1). Among these, Obstructive Fibrinous Tracheal Pseudomembrane (OFTP) is extremely rare; it serves as an uncommon cause of post-extubation stridor and represents a potentially life-threatening complication of endotracheal intubation. OFTP arises from relative ischemia of the tracheal wall, which is induced by overinflation of the endotracheal tube cuff or insufficient perfusion. It is characterized by the detachment of necrotic tracheal mucosa from the tracheal wall, forming a thick, tubular pseudomembrane. This condition may lead to varying degrees of stridor and dyspnea in affected patients (2). In previous reports, OFTP has been documented in both pediatric and adult populations. When detected and treated promptly, patients typically achieve favorable outcomes; however, fatal cases have indeed occurred among the reported instances (2, 3). Owing to the fact that current reports on OFTP are sporadic, it is challenging to determine its true incidence. Nevertheless, a study on pediatric OFTP conducted by Soong et al. (4) indicated that the incidence of OFTP among patients with post-extubation respiratory distress was 1.48%. This figure, however, may still be an underestimate, as some cases have not been investigated using flexible bronchoscopy (FB). Therefore, intensivists, pulmonologists, and otolaryngologists should remain vigilant regarding this relatively underrecognized complication of endotracheal intubation.

This case report describes a pediatric patient who developed acute airway obstruction due to the necrosis and detachment of OFTP following accidental self-extubation of the endotracheal tube after intubation. Additionally, it summarizes the patient's clinical features, diagnostic process, and treatment course.

## 2 Case report

A 10-year-old female pediatric patient was transferred directly from another hospital to the ICU of our hospital for further treatment due to poor therapeutic response 4 days after surgery for multiple injuries caused by a traffic accident. Upon admission to the ICU, the patient was conscious, presented with persistent stridor, and only complained of pain at the trauma site, with a Critical-Care Pain Observation Tool (CPOT) score of 3. She had no other medical history. Physical examination findings were as follows: body temperature 36.4 ℃, heart rate (HR) 106–130 beats/min, respiratory rate (RR) 16–20 breaths/min, pulse oxygen saturation (SPO_2_, under non-invasive ventilator-assisted ventilation) 96%–100%, and arterial blood pressure (ABP) 92/58 mmHg–146/73 mmHg. The Acute Physiology and Chronic Health Evaluation II (APACHE II) score and Sequential Organ Failure Assessment (SOFA) score of the patient at ICU admission were 15 and 10, respectively. At admission, the patient was diagnosed with: hemorrhagic shock; right femoral fracture, left foot skin avulsion injury, multiple pelvic fractures, and incomplete dislocation of lumbosacral vertebrae; female pelvic hematoma; and cervical tracheal injury.

**Figure 1:**
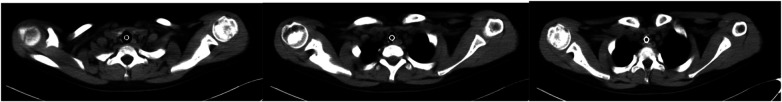
Tracheal condition of the patient before transfer to the ICU (the tracheal tube fits very tightly against the tracheal wall, suggesting that the diameter of the endotracheal tube may have been excessively large. This could be one of the reasons for airway injury following accidental extubation.).

Prior to admission to our ICU, the patient had a history of accidental endotracheal extubation. The detailed process was as follows: The patient required surgery due to multiple injuries. To ensure the patient's ventilation safety and meet surgical treatment needs, tracheal intubation was therefore used to assist with ventilation. After 3 days of endotracheal intubation, the patient experienced changes in comfort and pain levels, and she self-extubated the endotracheal tube while the tube cuff was fully inflated. Computed tomography (CT) findings are shown in Figure 1.

ICU day 1 (the first day after the patient was transferred to the ICU of our hospital): We observed that when the patient was on non-invasive ventilator-assisted ventilation in a quiet state, there was obvious stridor. The stridor persisted after intravenous push of dexamethasone, accompanied by inspiratory dyspnea, and the oxygen saturation dropped to 86%. Based on the symptoms, we suspected that the patient had glottic edema and planned to perform endotracheal intubation for assisted ventilation. During the intubation process, laryngoscopy revealed that the patient's epiglottis, glottis, and pharyngeal wall tissues were reddish, but no obvious edematous stenosis or airway compression was found. Even after fully exposing the upper respiratory tract, obvious stridor could still be heard from the patient. Meanwhile, a suspected white foreign body was visible in the airway below the glottis. At this point, the possibility of tracheal ring fracture and collapse could not be ruled out. To avoid airway injury caused by endotracheal intubation and the possibility of further inhalation of the foreign body, the intubation was suspended. The patient continued to receive non-invasive ventilator-assisted ventilation, along with nebulized inhalation of a bronchodilator (salbutamol) as adjuvant therapy.

**Figure 2:**
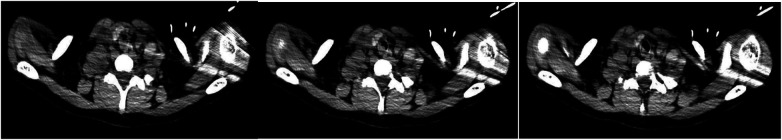
Tracheal condition of the patient on ICU Day 2.

ICU day 2: To examine the patient's airway condition, we performed a bedside fiberoptic bronchoscopy, which showed edema of the airway wall tissues, mucosal congestion (with easy bleeding upon touch), and a coin-shaped stenosis in the center of the airway. At this point, the fiberoptic bronchoscope could not proceed further downward. Subsequent CT showed tracheal stenosis (Figure 2). Methylprednisolone sodium succinate 40 mg was administered intravenously to reduce edema. Since the patient's vital signs were stable, without cough or expectoration, and the dyspnea did not deteriorate further, no further tracheotomy was performed. The patient continued to receive salbutamol nebulization and intravenous drip of methylprednisolone sodium succinate.

**Figure 3:**
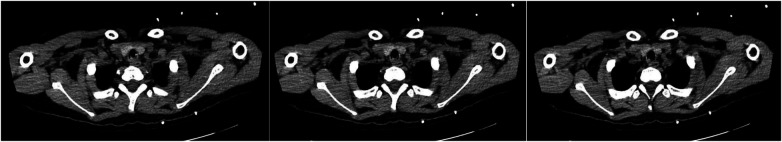
Tracheal condition of the patient before fiberoptic bronchoscopic debridement on ICU Day 4.

ICU day 4: The patient still had stridor and dyspnea. Auscultation of both lungs revealed obvious dry rales near the airway. Neck CT showed a local stenosis of the tracheal lumen at approximately 31 mm from the glottis, with abnormal density shadows in the trachea, as shown in Figure 3. On the same day, a bedside fiberoptic bronchoscopy was performed, and after consultation with the Department of Respiratory Medicine and Department of Otorhinolaryngology-Head and Neck Surgery, it was considered that the patient had necrosis of the neck mucosal tissues, and the diagnosis of OFTP was made.

**Figure 4:**
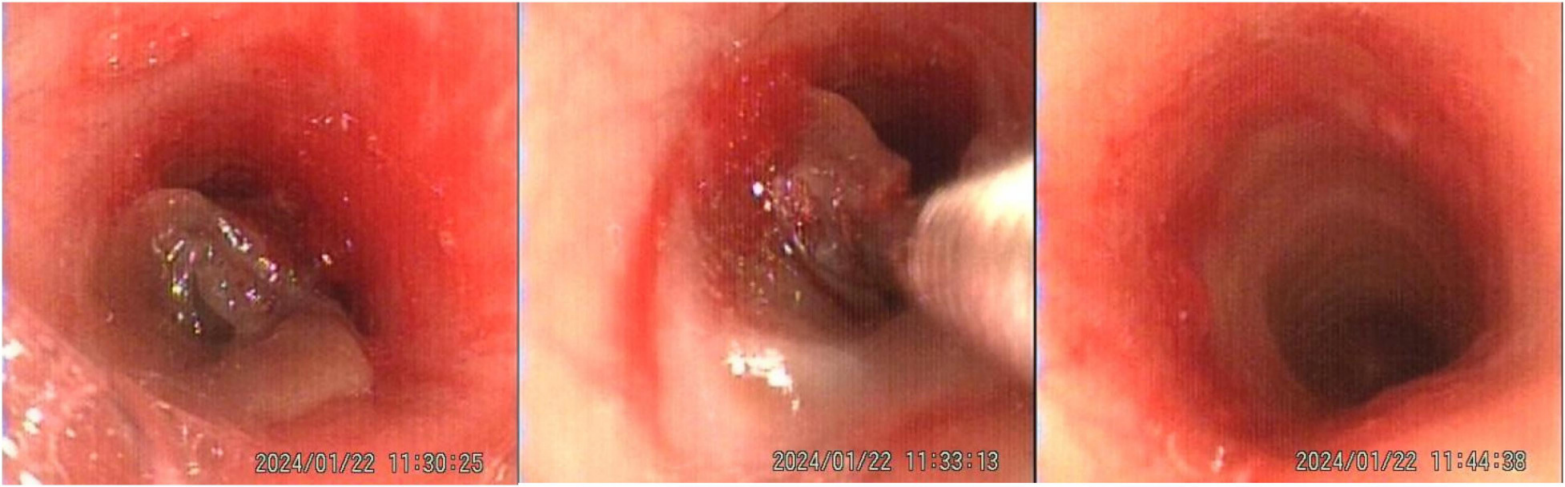
Tracheal condition of the patient during and after fiberoptic bronchoscopic debridement on ICU Day 5.

**Figure 5:**
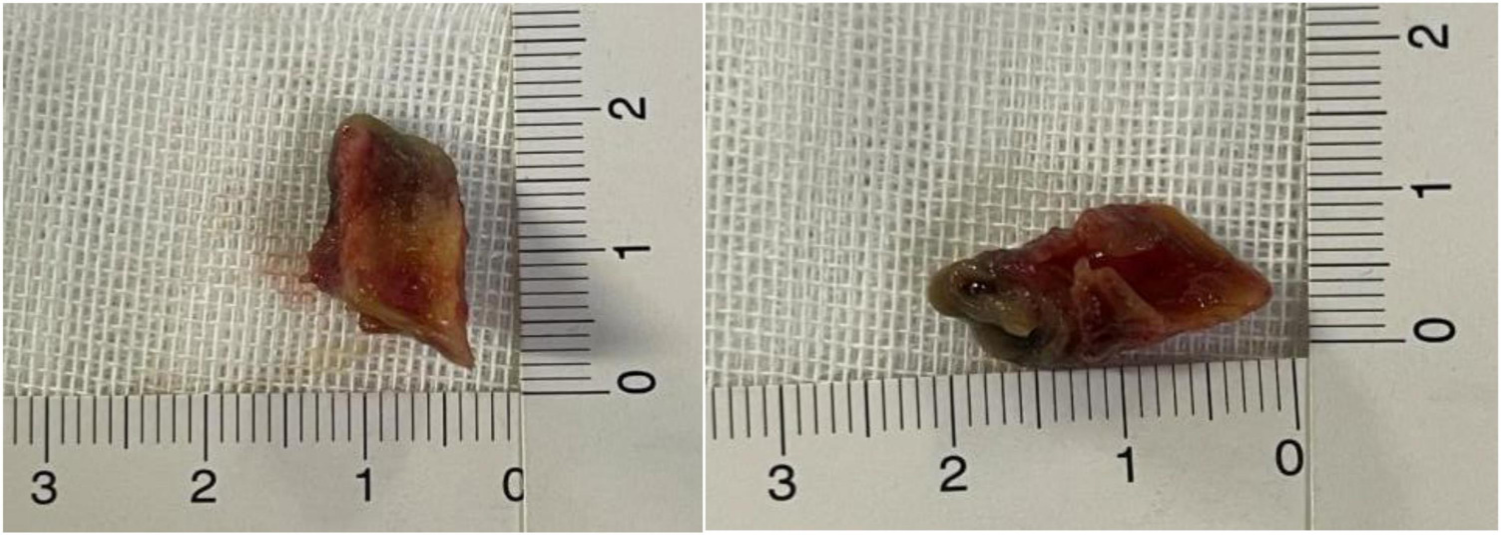
Stripped airway mucosal tissues of the patient on ICU Day 5.

ICU day 5: Mucosal necrotic tissue debridement was performed under fiberoptic bronchoscopy. Figure 4 shows the patient's tracheal condition during and after the debridement under fiberoptic bronchoscopy, and the debrided mucosal tissues are shown in Figure 5. After the removal of the necrotic airway mucosal tissues, the airway wall showed a red inflammatory response. The specific procedure is as follows:

After a thorough explanation of the patient's current condition and the purpose, procedures, results, and potential risks of fiberoptic bronchoscopy to the patient and her parents, both agreed to undergo the examination.

A multi-disciplinary team with rich experience conducted the fiberoptic bronchoscopy and treatment at the bedside of ICU. The team included anesthetists, respiratory therapists, respiratory physicians, ICU physicians, and two ICU nurses. The patient's heart rate, blood pressure, respiratory rate, and pulse oxygen saturation were continuously monitored. With the companionship of family members, the patient took a semi-recumbent position, receiving high-flow nasal cannula oxygen to maintain a pulse oxygen saturation above 96%. The anesthetist first performed local anesthesia in the nasopharynx and oropharynx, followed by titrating propofol and fentanyl to achieve a depth of sedation and analgesia with a RASS score of −2 to −1 and a CPOT score of 0–1. After adequately lubricating the tip of the fiberoptic bronchoscope, it was inserted through the right nostril. The throat, epiglottis, and glottis showed mild congestion. After supplementing local anesthesia at the glottis, the scope was advanced into the glottis, where about 3 cm below, a soft tissue obstructed the trachea, leaving only about one-third of the airway lumen available for gas passage. The soft tissue was mostly stripped off from the airway but still partially connected, forming a “flap”-like structure that exacerbated airway obstruction during expiration. Upon careful observation, no obvious bleeding was seen at the connection between the soft tissue and the airway mucosa. After evaluation by the multi-disciplinary team, they decided to use foreign-body forceps to attempt removal of this soft tissue and again obtained consent from the patient's family. Under the guidance of the fiberoptic bronchoscope, the foreign body forceps was inserted and after several attempts, the soft tissue was completely removed. A second examination of the airway under the fiberoptic bronchoscope revealed that the trachea had resumed patency. There was obvious congestion in the airway mucosa where the soft tissue adhered earlier, but there was no obvious bleeding on the surface or residual tissue. The fiberoptic bronchoscope was then used to further examine other airways and found a small amount of white sputum in the right lower lobe basal segmental bronchus, which was fully suctioned out. No abnormalities were found in other airways throughout the procedure lasting approximately 25 min. Throughout this inspection and treatment process, the patient's vital signs remained stable and she cooperated well. After cessation of anesthesia, the patient quickly regained consciousness and reported significant relief from dyspnea. She reported significant relief in her dyspnea symptoms and a marked decrease in throat discomfort. There was no significant sore throat or exacerbation of cough within 24 h postoperatively.

**Figure 6:**
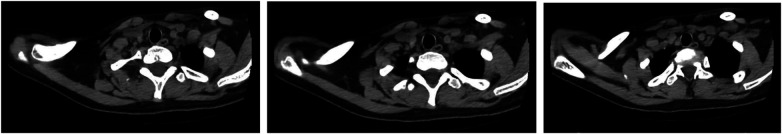
Re-examination of tracheal CT in the patient on ICU Day 8.

ICU day 8: A re-examination of the neck CT showed that the patient's airway was unobstructed with no abnormalities, as shown in Figure 6. The oxygen saturation was 100%.

The entire process is shown in the Supplementary Video.

Condition of the patient's other organs:

In terms of infection, due to mycoplasma infection, the lung CT showed large patches of ground-glass opacities with a small amount of consolidation in both lungs. On ICU Day 2, azithromycin was administered for anti-infection treatment, along with nebulization to resolve phlegm. On ICU Day 7, the patient's lung infection improved, and the infection indicators returned to normal, so the antibiotic treatment (azithromycin) was stopped.

In terms of the digestive system, the patient had gastrointestinal bloating upon admission to the ICU. After using lactulose for laxation and glycerin enema, the patient passed stool, and the abdomen became soft.

In terms of circulation, the patient had hemorrhagic shock upon admission to the ICU, with a decrease in hemoglobin. The patient's condition improved after transfusion of blood and fluid replacement. On ICU Day 8, the patient's vital signs were stable, with a hemoglobin level of 101 g/L, and the hemorrhagic shock was corrected. The patient was transferred to the general ward for further treatment. Eventually, the patient recovered and was discharged from the hospital.

## 3 Discussion

Airway obstruction caused by the detachment of OFTP due to tracheal mucosal injury from endotracheal intubation is extremely rare in clinical practice. Since the clinical manifestations of this disease are non-specific and its incidence is low, most clinicians have a low level of vigilance against it, making it difficult to immediately establish a clear diagnosis after symptoms appear. The detachment of damaged tracheal mucosa can form a valve-like obstruction in the trachea, leading to obvious stridor, acute respiratory distress, or even asphyxia, which may result in sudden death of the patient. Early clear diagnosis and proper management can help the patient turn the corner and recover smoothly.

The pathogenesis of OFTP remains unclear. It is generally believed that OFTP tends to occur in patients after endotracheal tube extubation. The cuff of the endotracheal tube compresses the tracheal wall, and when the cuff pressure exceeds 30 cmH_2_O (1 cmH_2_O = 0.098 kPa), it causes ischemic injury to the tracheal mucosa. Subsequently, submucosal hemorrhage and necrosis of the trachea occur, complicated by a severe inflammatory response, massive exudation of fibrinogen, and infiltration of neutrophils, ultimately leading to the formation of OFTP (5). A few scholars argue that OFTP does not necessarily occur predominantly in intubated patients. In adult patients, long-term smoking may also be an inducing factor for OFTP. Certain components in tobacco can chronically irritate the trachea, induce inflammatory reactions, and form pseudomembranes similar to OFTP on the tracheal wall (6). Additionally, research results indicate that the formation of OFTP may be related to aspiration of gastric contents and tracheal burns. The inflammatory reaction induced by chemical damage to the tracheal mucosa caused by gastric acid may be one of the reasons (7). Some studies have reported that bacteria, viruses, and fungi can also lead to the formation of OFTP (8). In this case, the cause of OFTP formation does not seem to be entirely one of the above-mentioned reasons. This case occurred because the endotracheal intubation caused discomfort to the child, and due to the poor self-control ability of children, the child forcibly extubated the tracheal tube when the cuff pressure was sufficient, resulting in tracheal mucosal injury and necrosis, and the formation of OFTP. However, there is also evidence that OFTP has occurred in patients with cuff pressure maintained below 25 cmH_2_O (9). Therefore, the pathogenesis of OFTP requires further exploration.

Through a literature review, it is found that the airway obstruction symptoms of OFTP can occur immediately, or they can appear after the mucosal tissue becomes necrotic and detaches, which may take several hours to a few days. The most common symptoms include hoarseness, stridor, or respiratory failure. Most of these symptoms are non-specific and can also be seen in laryngeal edema and/or spinal paralysis (5, 10). Due to the high possibility of missed diagnosis, patients usually receive supportive treatments such as nebulized inhalation of bronchodilators (salbutamol) and corticosteroids, or undergo reintubation before a correct diagnosis is made (11, 12), resulting in a long delay in the diagnosis of the disease. In a systematic review involving 24 subjects, 96.3% of them had symptoms, which usually appeared immediately after extubation. A systematic review by Sehgal et al. (13) reported 54 adult and pediatric patients. The median intubation duration for pediatric patients was 35 h, but the correct diagnosis of OFTP could be delayed until 70 days after extubation, and it was pointed out that the onset of OFTP symptoms was faster in their pediatric cases (14, 15). In other similar case reports, OFTP-related symptoms also appeared one day after tracheal extubation, but the correct diagnosis was made 10 days later (13). In another retrospective report on the occurrence of OFTP in children and neonates in the intensive care unit, it was pointed out that among the 8 enrolled OFTP patients, the incidence of respiratory distress after extubation was 1.48%, with an average intubation time of 37.6 ± 12.3 h; the average time from extubation to symptom onset was 3.6 ± 1.4 h, and the symptoms lasted from 20.8 to 20.3 h (4). It is worth noting that Rhame (16) described a case of OFTP in an infant who did not undergo prolonged intubation after laser surgery (intubation duration was 24 h). The infant had persistent stridor after extubation and died 15 h after discharge due to delayed diagnosis. In our case, the child was intubated for 3 days (72 h) and developed airway obstruction symptoms on the 2nd day after extubation. A definite diagnosis was made after fiberoptic bronchoscopy and multi-disciplinary consultations 4 days after the symptoms appeared, leading to a certain delay in the correct treatment. Fortunately, the condition did not worsen during this period. Studies have shown that there have been cases of adult deaths due to delayed diagnosis and treatment of OFTP (9). Therefore, OFTP is highly prone to missed diagnosis and misdiagnosis in clinical practice, which affects the patient's recovery.

In most cases, OFTP is treated with bronchoscopy and surgical resection (9). Flexible bronchoscopy is mainly used in pediatric cases or when rigid bronchoscopy is contraindicated (3). However, there are also reports that patients can spontaneously cough up tracheal necrotic substances or that the necrotic substances can be brought out during tracheal extubation (12). Previous literature has reported that in the treatment of OFTP, rigid bronchoscopes have an advantage over flexible bronchoscopes in that they can maintain continuous airway ventilation. Therefore, approximately 3/5 of patients have their tracheal necrotic substances directly removed via rigid bronchoscopes, and about 2/5 of patients have their tracheal necrotic substances cleared through flexible bronchoscopes (13). On the other hand, due to the loose texture of necrotic substances, they are fragile when grasped with biopsy forceps, and only a small portion can be taken each time. The operation takes a long time, and patients with severe tracheal obstruction are prone to the risk of asphyxia. In contrast, cryoablation can quickly clear tracheal necrotic substances, unblock the lumen, and is efficient and safe (17). In this case, since the patient was a child, the use of rigid bronchoscopy for treatment might further damage the patient's mucosa, so we adopted a softer flexible bronchoscope for treatment. Initially, because the necrotic annular pseudomembrane tissue was loose, only a small portion could be taken each time. Subsequently, by slowly stripping from the edge, we completely removed the entire piece of necrotic substance without causing damage or bleeding to the surface of the tracheal wall. The report and discussion of this case further confirm that flexible bronchoscopy is effective in treating similar pediatric cases and is a treatment method worthy of reference.

## 4 Limitation

In this case, we have to admit that there was a certain delay in the diagnosis of OFTP, which led to the misdiagnosis of the child's dyspnea symptoms and the failure to provide correct treatment. However, we found that for such cases, the initial conventional traditional treatment for stridor cannot improve the patient's symptoms, which provides a direction for the diagnosis of similar cases in the future. Moreover, case reports (18, 19) suggest that the traditional treatment for stridor may lead to sudden complete airway obstruction due to the emergency placement of an endotracheal tube. Therefore, for patients with laryngeal stridor of unknown cause, the treatment method should be determined according to the specific clinical situation.

## 5 Conclusion

OFTP caused by accidental extubation of endotracheal tube is a rare clinical condition that leads to acute inspiratory dyspnea. Flexible bronchoscopy is helpful for diagnosis and can non-invasively and quickly remove the necrotic and exfoliated tracheal mucosal tissues formed due to OFTP, thus relieving airway obstruction in pediatric patients. This case provides a new option for the diagnosis and treatment of similar patients in the future.

## Data Availability

The original contributions presented in the study are included in the article/Supplementary Material, further inquiries can be directed to the corresponding author/s.
